# Role of growth factors and oxygen to limit hypertrophy and impact of high magnetic nanoparticles dose during stem cell chondrogenesis

**DOI:** 10.1016/j.csbj.2018.10.014

**Published:** 2018-10-30

**Authors:** Aurore Van de Walle, Waïss Faissal, Claire Wilhelm, Nathalie Luciani

**Affiliations:** Laboratoire Matière et Systèmes Complexes (MSC), UMR 7057 CNRS, University Paris Diderot, 75205 Paris cedex 13, France

**Keywords:** Cartilage tissue engineering, Chondrogenesis, Mesenchymal stem cell, Hypoxia, Hypertrophy, Magnetic nanoparticles, Growth factors, ECM, extracellular matrix, MSC, mesenchymal stem cell, TGF-β, transforming growth factor beta, IGF-1, insulin-like growth factor 1, HIF, hypoxia inducible factor, NH, normoxic-hypoxic, RUNX2, runt-related transcription factor 2, MRI, magnetic resonance imaging

## Abstract

Due to an unmet clinical need of curative treatments for osteoarthritic patients, tissue engineering strategies that propose the development of cartilage tissue replacements from stem cells have emerged. Some of these strategies are based on the internalization of magnetic nanoparticles into stem cells to then initiate the chondrogenesis via magnetic compaction. A major difficulty is to drive the chondrogenic differentiation of the cells such as they produce an extracellular matrix free of hypertrophic collagen. An additional difficulty has to be overcome when nanoparticles are used, knowing that a high dose of nanoparticles can limit the chondrogenesis. We here propose a gene-based analysis of the effects of chemical factors (growth factors, hypoxia) on the chondrogenic differentiation of human mesenchymal stem cells both with and without nanoparticles. We focus on the synthesis of two of the most important constituents present in the cartilaginous extracellular matrix (Collagen II and Aggrecan) and on the expression of collagen X, the signature of hypertrophic cartilage, in order to provide a quantitative index of the type of cartilage produced (i.e. hyaline, hypertrophic). We demonstrate that by applying specific environmental conditions, gene expression can be directed toward the production of hyaline cartilage, with limited hypertrophy. Besides, a combination of the growth factors IGF-1, TGF-β3, with a hypoxic conditioning remarkably reduced the impact of high nanoparticles concentration.

## Graphical Abstract

**Figure:**
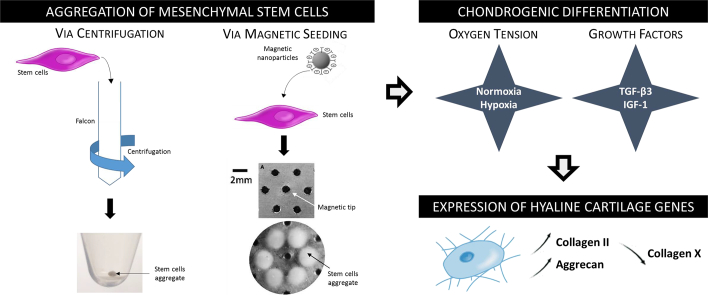

## 1 Introduction

Articular (hyaline) cartilage is an avascular tissue with a rich extracellular matrix and a low cell density and turnover. As a consequence, its repair remains a significant challenge. For patients with full-thickness articular defects, total joint replacement surgery is the most common procedure; however, postponing or avoiding it via treatment of the cartilage defect would be preferable. Various options exist including autologous chondrocyte implantation (ACI), osteochondral grafts, and microfracture, which have all shown various degrees of success but typically prove unsatisfactory in the long term or are linked to donor site morbidity [[1], [2], [3]]. For this reason, tissue engineering strategies that propose the development of cartilage tissue replacements have emerged. Mesenchymal stem cells (MSCs) present a high clinical potential as a cell source for the engineering of these cartilage tissues as they present long-term self-renewal ability, a higher proliferative turnover than differentiated chondrocytes, and can be extracted from patients opening up the possibility of developing autologous grafts [[4], [5], [6]].

When undergoing chondrogenesis, MSCs synthesize extracellular matrix (ECM) components that are in majority collagens, proteoglycans, and an assortment of glycoproteins [7,8]. The stoichiometry of the ECM assembly is critical and varies depending on the type of cartilage produced [9,10]. For articular (hyaline) cartilage, collagen II and aggrecan are two of the most important constituents: type II collagen constitutes >80% of the ECM, providing the cartilaginous structure and tensile strength, while aggrecan constitutes 80 to 90% of the proteoglycans and provide compressive strength as well as fluid regulation. Oppositely, collagen X expression is a marker of hypertrophic cartilage, which can result in ossification [11,12]. A major difficulty when using MSCs as a cell source for articular cartilage tissue engineering is to drive their chondrogenic differentiation such as they produce a hyaline extracellular matrix free of hypertrophic collagen [5,13].

The development, growth, and maintenance of articular cartilage are controlled by several very strict signaling pathways governed by the microenvironment. For example, cell–cell interactions similar to those observed in pre-cartilage condensations during embryogenesis are critical to initiate chondrogenesis. For this reason, pellet culture, which provides a three-dimensional environment, is a standard culture method for MSCs-based cartilage engineering [14]. Numerous bioactive growth factors also trigger cartilage differentiation and maintenance. In vivo*,* these growth factors are stored in the ECM and released under specific conditions, such as tissue damage. In vitro, they can be used alone or in combination to support differentiation processes; however, exact synergistic effects still need to be identified [15].

Within these growth factors are the members of the transforming growth factor beta (TGF-β) superfamily that have an early positive role in cartilage development by enhancing ECM synthesis and by inducing functional and long-term differentiation of stem cells [16]. Of the three isoforms (TGF-β1, TGF-β2, and TGF-β3), most investigators have used either TGF-β1 or TGF-β3 [17,18]. Indeed, TGF*-*β*2 might not be the most suited to obtain hyaline cartilage as its* highest expression levels are in the hypertrophic zone of the cartilage growth plate [19,20]. Studies reported TGF-β3 to have a higher chondrogenic potential than TGF-β1 and to lead to a more rapid differentiation [18]. Moreover, the faster and stronger differentiation initiated by TGF-β3 is not associated with a higher susceptibility for hypertrophy [21]. On the contrary, it has been indicated that terminal differentiation in vitro is most likely repressed by TGF-β3, which retains chondrocytes in the pre-hypertrophic state [17,21]. We herein intend to initiate chondrogenesis via the use of TGF-β3 and to assess the effects of various supplementation patterns. Insulin-like growth factor 1 (IGF-1) is another growth factor considered to have a major role in articular cartilage as it induces a plethora of anabolic effects and decreases catabolic responses. Among its positive characteristics, IGF-1 at a concentration of 100 ng/mL has been shown to maintain the chondrocyte phenotype and prevent cells from progressing to the hypertrophic stage [22]. As a combination of factors might be beneficial for cartilage engineering, we here assessed synergistic effects of TGF-β3 and IGF-1.

In addition to growth factors, oxygen tension plays an important role in controlling the chondrogenic differentiation of MSCs. Healthy articular cartilage is an avascular tissue in which oxygen is supplied to the chondrocytes by diffusion from the synovial fluid. Oxygen supply is then limited, with tension ranging from 7% O_2_ at the surface to 1% O_2_ in the deep zones [23,24]. Chondrocytes respond to this natural hypoxic environment via regulation of hypoxia inducible factor (HIF) family members (HIF-1α, -2α, -3α). In cartilage, HIF-1α has been determined as essential for chondrocyte growth arrest and survival [25]. Hypoxia has also been shown to enhance the chondrogenic differentiation of MSCs [26], while simultaneously impeding their hypertrophic differentiation with collagen X expression being inhibited via down-regulation of runt-related transcription factor 2 (Runx2) activity [27,28]. For this reason, the effect of growth factors supplementation will here be assessed under both normoxia and hypoxia.

Magnetic nanoparticles have attracted increasing attention for the repair of cartilage defects. Indeed, they offer a range of opportunities such as the magnetic targeting of MSCs at articular defect site, or again the tracking of engineered cartilage upon implantation using magnetic resonance imaging (MRI) [29,30]. They also present a clear advantage for tissue development as, upon nanoparticles' internalization, cells can be magnetically manipulated at a distance [31]. It is then possible to compact cells within or without scaffolds, which is an essential step to initiate chondrogenesis, and to further produce cm-scale tissues mimicking cartilage at best [[32], [33], [34]]. Within weeks following internalization, magnetic nanoparticles have been shown to be progressively degraded when stem cells are cultured under a chondrogenic model [35,36]. The iron released by their degradation has no impact when low doses of nanoparticles are internalized (≤ 10 pg iron/cell) with the released iron integrating the native iron metabolism of the organism and being stored in the ferritin [35,37]. However, at a high nanoparticles dose (> 30 pg iron/cell), the important release of iron has a negative impact on chondrogenesis, most probably due to an excess of iron ions in the cells driving the production of reactive oxygen species (ROS) generated by the Fenton reaction [38,39]. As a consequence, the production of collagen II and aggrecan is reduced [38]. Interestingly, other differentiation pathways of stem cells, such as adipogenesis and osteogenesis, are not impacted by similarly high doses of nanoparticles [38,40]. In an effort of trying to reduce the negative impact of a high dose of nanoparticles on chondrogenesis, we aimed here to investigate if a fine control of the microenvironment can have positive effects on nanoparticles' labeled stem cells.

**Fig. 1:**
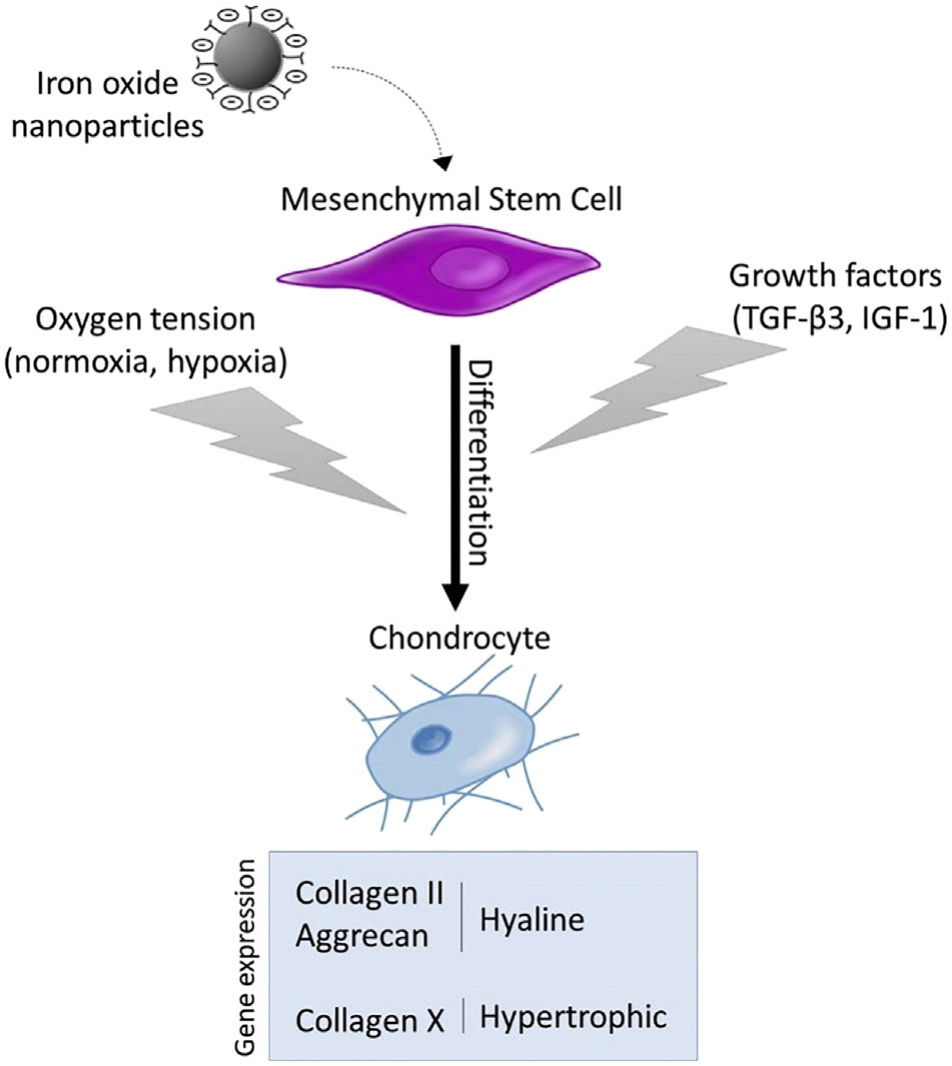
Schematic of the culture parameters assessed (e.g. growth factors, oxygen tension) on chondrogenic differentiation of mesenchymal stem cells, labeled or not with magnetic nanoparticles. In this paper, effects of two growth factors (TGF-β3 and IGF-1) as well as oxgen tension (normoxia and hypoxia) on the expression of three genes is studied. The expression kinetics of collagen II and aggrecan are assessed as markers of hyaline cartilage, while collagen X as a marker of hypertrophic cartilage. By varying culture parameters we assess if the gene expression of hyaline cartilage markers can be stimulated and if deleterious effects, observed when high doses of magnetic nanoparticles (composed of iron oxide) are internalized, can be avoided.

We propose a gene-based analysis to investigate whether exogenous IGF-1 and TGF-β3 as well as oxygen tension, separately and in combination, can enhance expression of hyaline cartilage molecular markers in human MSCs, labeled or not with magnetic nanoparticles (Fig. 1). We focus on the expression of collagen II and aggrecan - the most important components present in hyaline cartilage - and on the expression of collagen X - signature of hypertrophic cartilage - in order to provide a quantitative index of the type of cartilage produced (i.e. hyaline, hypertrophic). We here demonstrate a clear positive impact of culture under a combination of TGF-β3, IGF-1, and hypoxia to both drive the chondrogenic differentiation toward hyaline cartilage and to counter potential negative effects linked to high doses of nanoparticles' labeling.

## 2 Material and Methods

A materials list is included as Table S1.

### 2.1 Magnetic Nanoparticles

Iron oxide (maghemite) nanoparticles were synthetized by coprecipitation of iron salts, and chelated with citric acid. The stability of the colloidal suspension is ensured by electrostatic repulsion thanks to the negative charges brought by the citrate carboxylate groups (COO-). The magnetic nanoparticles obtained exhibit a typical superparamagnetic behavior and present a mean diameter of 8 nm (polydispersity = 0.35).

### 2.2 Cell Culture and Nanoparticle Labeling

Human bone marrow-derived mesenchymal stem cells isolated from the posterior iliac crests of normal adult volunteers (Poietics, Lonza) were cultured in MSCGM medium (Lonza) at 37 °C with 5% CO_2_. These cells are positive at >99% for the markers CD105, CD166, CD44, CD90, and CD73. Cells were grown until 80% confluence prior to labeling. A labeling solution was prepared with nanoparticles diluted at a concentration of [Fe] = 0.1 mM (low dose) or 0.8 mM (high dose) in serum free RPMI culture medium (Thermo) supplemented with 5 mM free citrate (to avoid nanoparticles' precipitation). Cells were labeled via incubation with this solution at 37 °C for 30 min, time for the nanoparticles to be internalized. Cells were then rinsed thoroughly in serum-free RPMI medium and incubated for 4 h with complete MSCGM medium before further processing. The iron load per cells was quantified by vibrating sample magnetometry.

### 2.3 Cell Differentiation

For chondrogenic differentiation, MSC (labeled or unlabeled) were detached with trypsin, centrifugated, and resuspended in the chondrogenesis medium at very high cell density. Cells were then either centrifuged to form a pellet of 2.5 × 10^5^ cells or seeded magnetically. For the magnetic seeding, cells were seeded over magnetic devices consisting of 750 μm in diameter magnetic tips overlying a permanent neodymium magnet; which ensured magnetization to saturation. Glass-bottomed cell culture dishes (35 mm) were placed on the top of these devices, and spheroid aggregates of 2.5 × 10^5^ cells were formed in chondrogenesis medium as previously described [32]. The aggregates were left on the device for at least 3 days, before being transferred into a 12-wells plate. The chondrogenic medium was made of serum-free high glucose DMEM with 1% penicillin-streptomycin, 0.1 μM dexamethasone, 1 mM sodium pyruvate, 50 μM l-ascorbic acid 2-phosphate, 0.35 mM L-proline (Sigma-Aldrich), 1% ITS-Premix (Corning). To study the effects of growth factors addition patterns on the chondrogenic differentiation, this chondrogenic medium was supplemented with the growth factors described below. The medium was replenished twice a week. After 7, 14, 20, and 27 days of differentiation, pellets were crushed and RNA was extracted. At day 27, spheroids were also fixed in 10% formalin, cryosectioned and stained with toluidine blue (0.5%) for 2 min at room temperature (Sigma–Aldrich, Saint-Quentin Fallavier, France) to detect glycosaminoglycans.

### 2.4 Growth Factors Supplementation Patterns and Oxygen Conditions

**Fig. 2:**
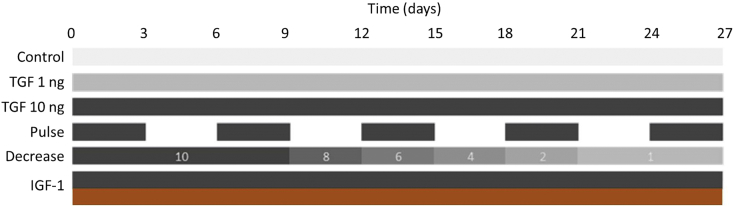
Supplementation patterns of studied growth factors. The impact of varying patterns of growth factors on the quality of chondrogenesis is assessed. A low concentration of TGF-β3 at 1 ng/mL (TGF 1 ng) is first compared to a more standard concentration at 10 ng/mL (TGF 10 ng). Pulses of TGF-β3 are then performed (Pulse), with TGF-β3 present in the culture media for three days then absent for three days all along the differentiation. A concentration of TGF-β3 going from 10 ng/mL at day 0 and progressively decreasing down to 1 ng/mL at day 27 is also assessed (Decrease). Last, a combination of TGF-β3 at 10 ng/mL and IGF-1 at 100 ng/mL is studied (IGF-1).

The chondrogenic differentiation medium was freshly prepared as described above. Negative control aggregates were cultured in chondrogenic medium without growth factors under normoxia. Various growth factors supplementation patterns were investigated as follows (see Fig. 2 for representative schematic). TGF 1 ng, for which the chondrogenic medium was continuously supplemented with 1 ng/mL of TGF-β3 (Interchim). TGF 10 ng, for which the chondrogenic medium was continuously supplemented with 10 ng/mL of TGF-β3. Pulse, for which TGF-β3 (10 ng/mL) was added for three days then removed for three days during the 27 days of culture. Decrease, for which a decreasing concentration of TGF-β3 going from 10 ng/mL to 1 ng/mL was added. IGF-1, for which the chondrogenic medium was continuously supplemented with 10 ng/mL of TGF-β3 combined with 100 ng/mL of IGF-1. The effects of these growth factors kinetics were studied under various oxygen tension, meaning under normoxia (21% oxygen) and hypoxia (3% oxygen). For hypoxia, a specific incubator (INCO 246med, Memmert) was used, in which the oxygen concentration can be decreased down to 3% by displacing oxygen with nitrogen.

### 2.5 RNA Isolation, cDNA Synthesis, and Quantitative RT-PCR

Total RNA from differentiated or undifferentiated cells with or without magnetic labeling was prepared using the total RNA isolation kit (Machery-Nagel) that includes a DNase treatment to avoid contamination with genomic DNA. Complementary DNA (cDNA) was synthesized using SuperScript II Reverse Transcriptase (Invitrogen). Real-time quantitative polymerase chain reaction was performed in duplicate with the StepOnePlus detection system (Thermo Fisher Scientific) and SYBRGreen dye (Applied Biosystems) according to the manufacturer's protocol. Levels of genes of interest mRNA were normalized to the housekeeping gene ribosomal protein large subunit P0 (RPLP0). Gene expression at day 0 was used as the control. Amplification of specific transcripts was confirmed by melting curves profiles generated at the end of the PCR program. The fluorescence cycle threshold (Ct) was calculated to quantify the relative gene expression.

Briefly, mRNA levels of the genes of interest (I) were expressed relative to levels of RPLP0 mRNA.

(ΔCt = Ct_I_ - Ct_RPLP0_) and the relative amount of mRNA levels between cells cultured under specific conditions (i.e. growth factors, hypoxia) and cells cultured without growth factor supplement under normoxia (control) is given by 2^−ΔΔCt^, where ΔΔCt = [ΔCt_I_ of culture condition of interest] – [ΔCt_I_ cells cultured without growth factors under normoxia]. See Table S2 for primer sequences.

### 2.6 Transmission Electron Microscopy (TEM)

Spheroids harvested at day 27 were rinsed and fixed with 5% glutaraldehyde in 0.1 mol/L sodium cacodylate buffer, and post-fixed with 1% osmium tetroxide solution containing 1.5% potassium cyanoferrate. The spheroids were then gradually dehydrated in ascending concentrations of ethanol and embedded in Epon resin. Thin sections (70 nm) were examined with a Zeiss EM 902 transmission electron microscope at 80 kV (MIMA2 platform, INRA, Jouy-en-Josas, France).

### 2.7 Statistical Analysis

All values are presented as mean ± standard error of the mean (SEM). Significance between groups was determined using independent Student's *t*-test. For all values, a minimum of 95% confidence level was considered significant, with * *p* < .05, ** *p* < .01 and *** *p* < .001. Number of independent measurements was systematically superior to 3 (*n* > 3).

## 3 Results

### 3.1 Impact of TGF-β3 and IGF-1 Supplementation on Chondrogenesis Kinetics under Normoxia

**Fig. 3:**
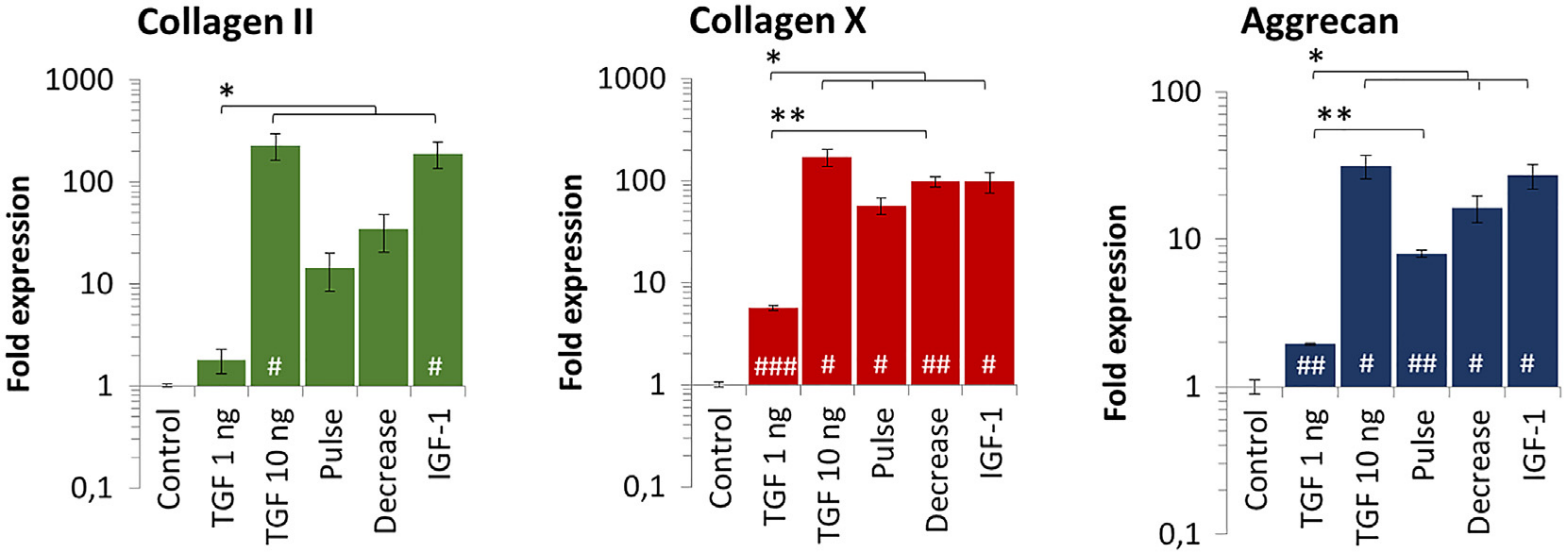
Impact of growth factors (TGF-β3 and IGF-1) supplementation on gene expression of collagen II, aggrecan, and collagen X after 27 days of chondrogenic differentiation (under normoxia). A continuous concentration of TGF-β3 at 1 ng/mL is too low to sufficiently drive chondrogenic differentiation. On the other hand, a continuous supplementation of TGF-β3 at 10 ng/mL drives highest expression of the hyaline cartilage markers collagen II and aggrecan. However, collagen X, markers of hypertrophic cartilage, is also highly expressed. A combination of both TGF-β3 (10 ng/mL) and IGF-1 (100 ng/mL) didn't induce a synergistic effect as results are similar to the 10 ng/mL TGF-β3 condition. Expression was normalized to RPLP0 mRNA and expressed relative to average value of cells cultured without growth factor supplement (control). Significance between groups was determined using independent Student's *t*-test. # represents significant differences when compared to the control. * represents significant differences between conditions.

**Fig. 4:**
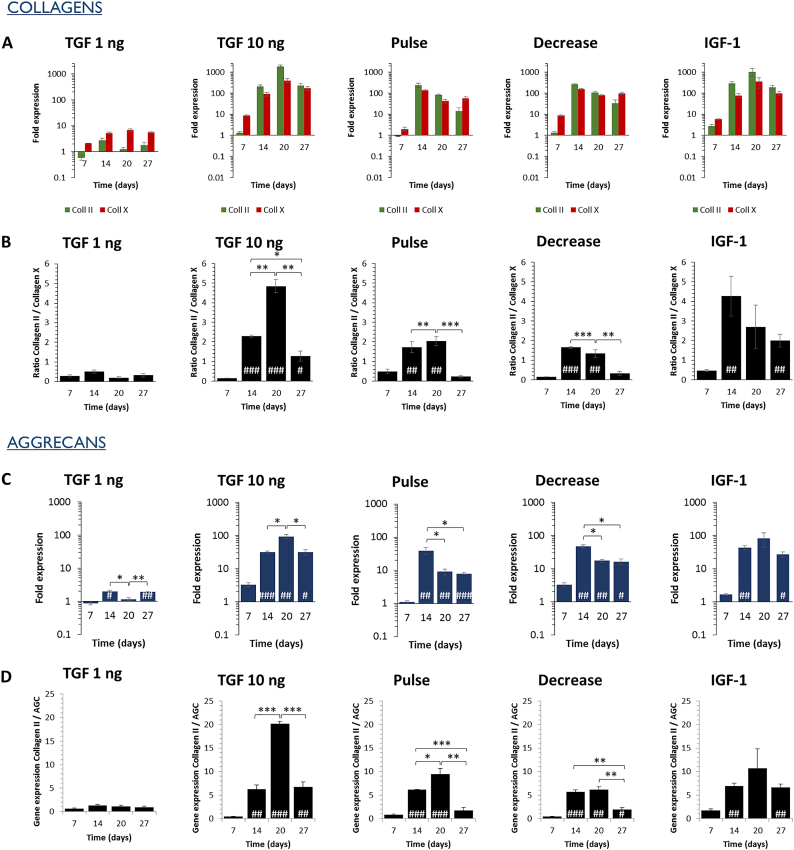
Varying growth factors supplementation patterns influence the kinetics of ECM components' gene expression under normoxia. A 27-days chondrogenic differentiation is induced under normoxia using varying growth factors supplementation patterns, described in Fig. 2. (A) The gene expression of collagen II as well as collagen X is assessed and their statistical significance is displayed in Table S3 (B) The collagen II-to-collagen X ratio is calculated and used as an indicator of hypertrophy level. (C) Aggrecan gene expression, typical ECM component of hyaline cartilage is assessed. (D) The collagen II-to-aggrecan ratio is calculated to ensure that an important secretion of aggrecan does not have a negative impact on the expression of collagen II. Expression was normalized to RPLP0 mRNA and expressed relative to average value of cells cultured without growth factor supplement (control). Significance between groups was determined using independent Student's *t*-test. # represents significant differences when compared to the control. * represents significant differences between conditions.

As chondrogenesis in vitro is typically performed under normoxia (21% O_2_), the impact of the growth factors TGF-β3 and IGF-1 on the expression kinetics of three genes (collagen II, collagen X, and aggrecan) as well as their ratios (collagen II/collagen X, collagen II/aggrecan) was first assessed under normoxia. The standard differentiation protocol using TGF-β3 consists in a continuous supplementation at a concentration of 10 ng/mL [41]. However, it has been suggested that a lower dose of TGF or alternative supplementation patterns might limit hypertrophy and improve glycosaminoglycan's synthesis [41]. We thus assessed a minimal dose of TGF-β3 (1 ng/mL) as well as diverse patterns such as pulses of TGF-β3 (10 ng/mL added every other media change), and a progressively decreasing dose of TGF-β3 (from 10 ng/mL at day 0 to 1 ng/mL at day 27). All conditions are expressed relatively to the cells cultured without growth factors (control) (Fig. 2). We here showed that a concentration above 1 ng/mL is needed to achieve chondrogenesis (Fig. 3). Indeed, in Fig. 4 it is demonstrated that a concentration at 1 ng/mL is too low to efficiently initiate chondrogenesis as collagen II is not expressed and aggrecan remains significantly lower than in all other conditions (Fig. 4 A, C). Moreover, under this low TGF-β3 concentration, collagen X expression is higher than collagen II leading to poor collagen II-to-collagen X ratios that are indicative of hypertrophy (Fig. 4 B). Interestingly, the standard culture protocol consisting of a continuous add of TGF-β3 at 10 ng/mL (condition named TGF 10 ng) gave the best results with the highest collagen type II and aggrecan expression. This high expression was reflected structurally as aggregates cultured for 27 days under TGF 10 ng and stained with toluidine blue displayed the characteristic blue color indicative of proteoglycans occurrence, which was not observed in the negative control (Fig. S1 A and B). Similarly conditioned aggregates observed in transmission electron microscopy also clearly demonstrated an extensive production of ECM and the presence of an important amount of collagen type II, recognizable by the typical periodicity of the fibers, while no ECM production was observed in the negative control (Fig. S1 C and D).

When observing the kinetics of collagen II and aggrecan expression for the TGF 10 ng condition, we can see that the expression is the highest at day 20 (Fig. 4 A, C). Moreover, the highest collagen II-to-collagen X ratio is observed at day 20,indicating that the hypertrophy is weaker than at day 27 (Fig. 4B). Similarly, collagen II-to-aggrecan was the highest at day 20 (Fig. 4D). The other TGF-β3 conditions gave no additional benefits under normoxia.

IGF-1 is a growth factor playing a major role in cartilage; its effects when in combination with TGF-β3 are here studied. We thus assessed the synergistic effects of IGF-1 at 100 ng/mL supplemented to the standard TGF 10 ng condition described above. We observed that under normoxia, the extra add of IGF-1 gave no additional benefits regarding to collagen II and aggrecan expression as well as collagen II-to-collagenX and collagen II-to-aggrecan ratios (Fig. 4).

### 3.2 Impact of Hypoxia

In the native environment, the cartilage tissue is subjected to a transition from normoxia to hypoxia over its development. We performed initial in vitro experiments to assess effects of a transitional normoxia-hypoxia (NH) conditioning by comparison to continuous normoxia (N) or hypoxia (H). For the NH culture, cells were placed under normoxia (21% O_2_) for 7 days followed by hypoxia (3% O_2_) for 20 days. Aggregates cultured for 27 days under this NH conditioning displayed an increasing trend in collagen II and aggrecan expression when compared to continuous normoxia (see Fig. S2). More interestingly, when compared to continuous hypoxia, they presented an increased trend in collagen II and a significant increase in aggrecan, while no difference in collagen X. These results demonstrate that NH conditioning increases gene expression of cartilage ECM components more efficiently than a continuous hypoxic culture. The effects of TGF-β3 and IGF-1 supplementation dynamics mentioned above were thus assessed under 7 days of normoxia followed by 20 days of hypoxia.

**Fig. 5:**
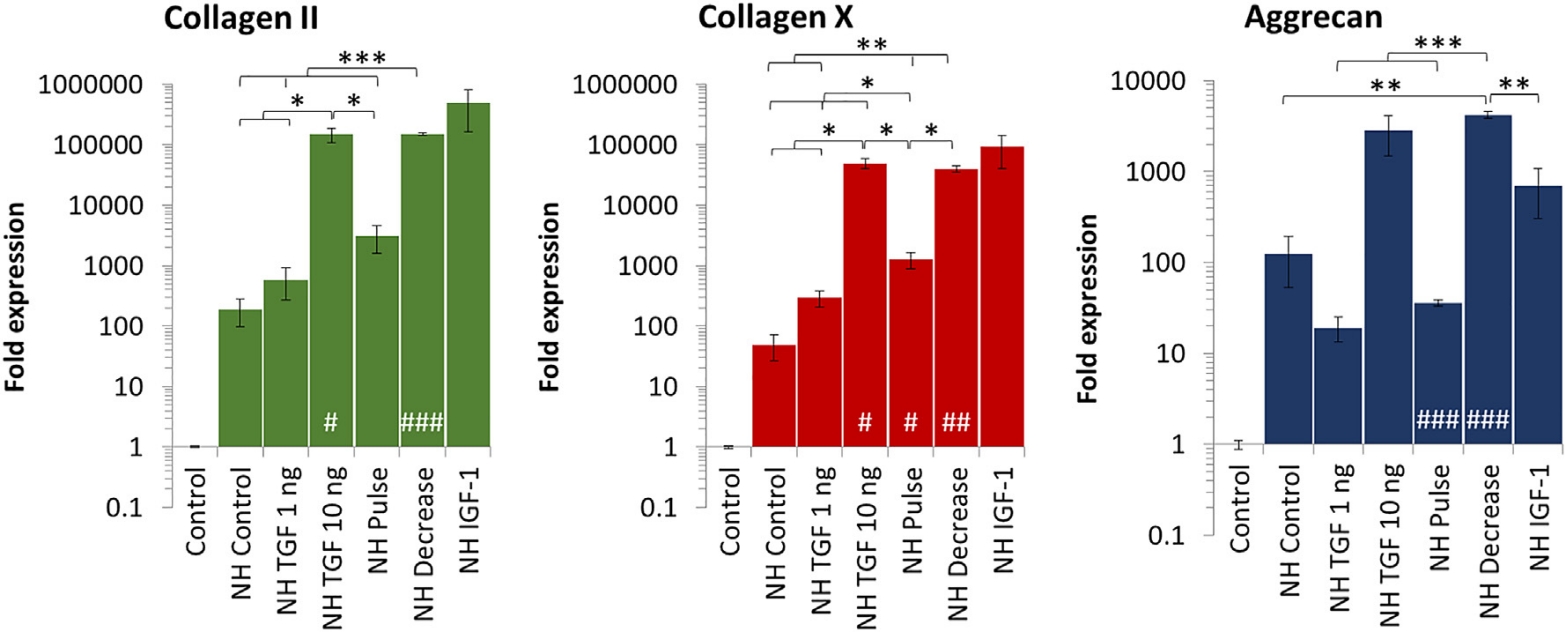
Hypoxia as a tool for chondrogenesis improvement. An initial 7-days culture period under normoxia (21% O_2_) is followed by 20 days under hypoxia (3% O_2_) and gene expression is assessed at day 27. This normoxia-hypoxia (NH) culture conditioning increases the expression of collagen II, collagen X, and aggrecan even without the use of growth factors (condition named NH Control). The effect is amplified in presence of TGF-β3 and IGF-1. Expression was normalized to RPLP0 mRNA and expressed relative to average value of cells cultured without growth factor supplement under normoxia (control). Significance between groups was determined using independent Student's *t*-test. # represents significant differences when compared to the control. * represents significant differences between conditions.

**Fig. 6:**
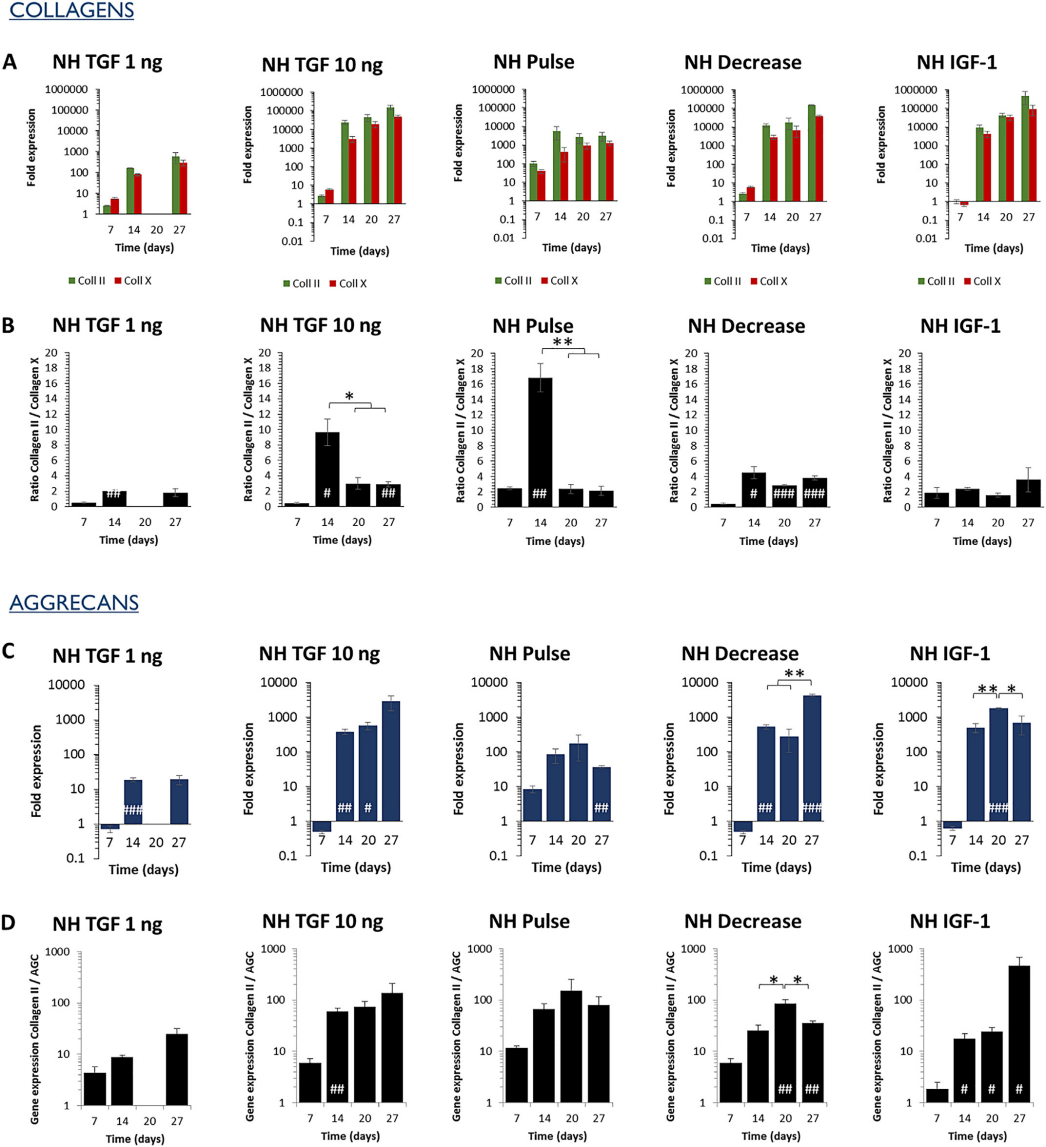
Varying growth factors supplementation patterns and hypoxic culture set-up influence the kinetics of ECM components' gene expression. The chondrogenic differentiation of MSCs is induced for 27 days using varying growth factors supplementation patterns (described in Fig. 2) and normoxic-hypoxic (NH) culture, with 7 days under normoxia followed by 20 days under hypoxia. (A) The gene expression of collagen II as well as collagen X is assessed and their statistical significance is displayed in Table S4. (B) The collagen II-to-collagen X ratio is calculated and used as an indicator of hypertrophy level. (C) Aggrecan gene expression, typical ECM component of hyaline cartilage is assessed. (D) The collagen II-to-aggrecan ratio is calculated to ensure that an important secretion of aggrecan does not have a negative impact on the expression of collagen II. For TGF 1 ng at day 20, the data is missing. Expression was normalized to RPLP0 mRNA and expressed relative to average value of cells cultured without growth factor supplement under normoxia (control). Significance between groups was determined using independent Student's *t*-test. # represents significant differences when compared to the control. * represents significant differences between conditions.

First result worth noticing is that NH conditioning led to a 190-fold increase in collagen II and a 123-fold increase in aggrecan expression at day 27 without any growth factor present in the culture media (Fig. 5). This increase was also present but lessened for collagen X (48-fold). This influence of NH is amplified when growth factors are added. Indeed, gene expression kinetics at day 7, 14, 20, and 27 show a higher expression of hyaline ECM components for all conditions and time points as shown by a significant increase in collagen and aggrecan when compared to normoxia (Fig. 6 A, C). The expression of collagen X was also increased (Fig. 6 A), but collagen II-to-collagen X ratios were improved under hypoxia compared with normoxia; with a maximum at 10 under NH with TGF-β3 at 10 ng/mL (day 14), while at 5 under normoxia with TGF-β3 at 10 ng/mL (day 20) (Fig. 6B). Collagen II-to-aggrecan ratios were also higher with a value at 61 under NH with TGF 10 ng (day 14), while at 20 under normoxia with TGF 10 ng (day 20) (Fig. 6D) demonstrating that increased aggrecan expression didn't impede collagen II synthesis.

When comparing the various growth factors supplementation patterns, the continuous presence of TGF-β3 at 10 ng/mL gave interesting results as it led to high expression of collagen II and aggrecan as well as high collagen II-to-collagen X (particularly at day 14) and collagen II-to-aggrecan ratios. A high collagen II-to-collagen X ratio was also observed at day 14 for the pulses of TGF-β3. However, the overall collagen II expression was higher for the continuous supplementation of TGF-β3 at 10 ng/mL than for the pulses. A progressive decrease of TGF-β3 (NH Decrease) also gave interesting results, with collagen II and aggrecan increasing in a similar way than with a continuous TGF-β3 supplementation (NH TGF 10 ng). In contrast, under normoxia the decrease of TGF-β3 led to decreased collagen II and aggrecan expression. In such case, the differentiation seems sufficiently advanced for chondrocyte maintenance and expression of hyaline markers until day 27. Finally, the impact of IGF-1 supplementation was limited with a low and late impact on collagen II expression, slightly higher only at day 27.

### 3.3 Counter Negative Effects Linked to High Nanoparticle Doses

**Fig. 7:**
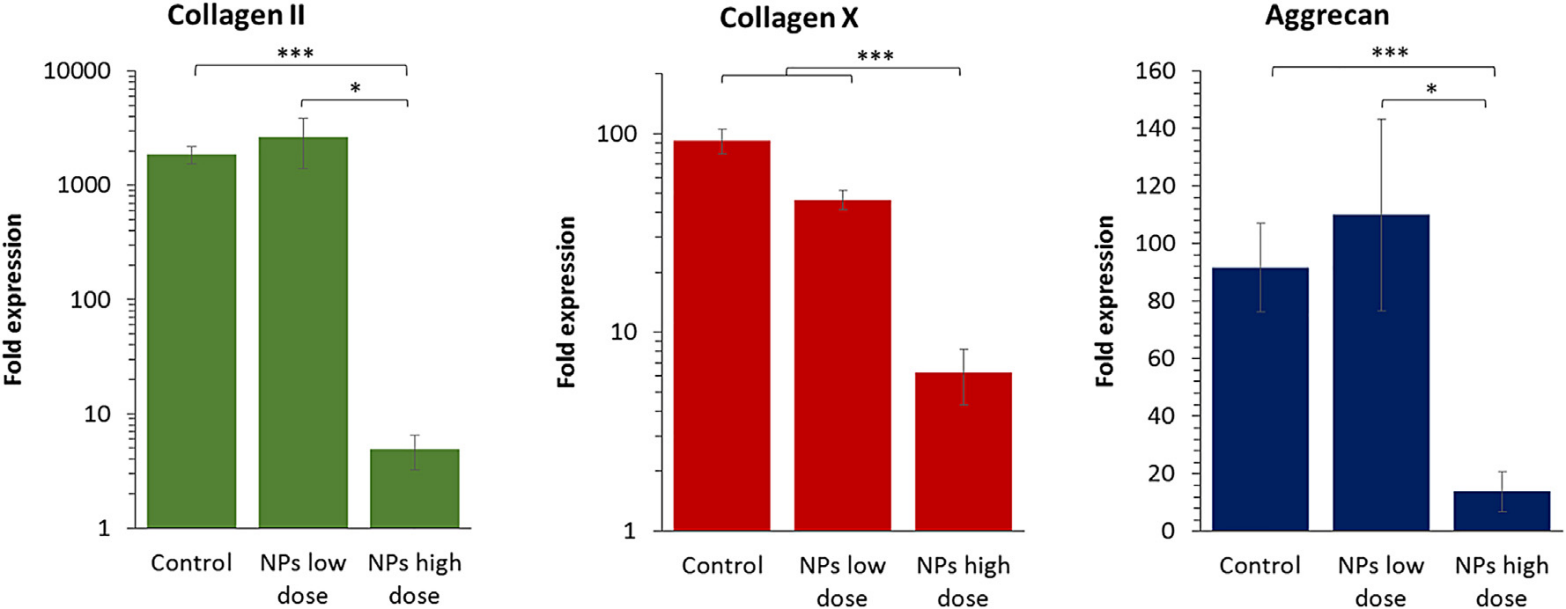
A dose-dependent impact of iron oxide nanoparticles on chondrogenesis. Nanoparticles are internalized in stem cells at day 0, cells are then differentiated for 20 days under a continuous supplementation of TGF-β3 at 10 ng/mL under normoxia. When a low dose (≤ 10 pg/cell) of nanoparticles is internalized in the cells, their chondrogenic differentiation capacity remains intact, with similar gene expression than unlabeled cells. However, when a high dose is internalized (> 30 pg/cell), synthesis of ECM components collagen II and aggrecan, but also collagen X is significantly reduced. Expression was normalized to RPLP0 mRNA and expressed relative to average value of unlabeled cells cultured without growth factor supplement under normoxia (control). Significance between groups was determined using independent Student's *t*-test. # represents significant differences when compared to the control. * represents significant differences between conditions.

**Fig. 8:**
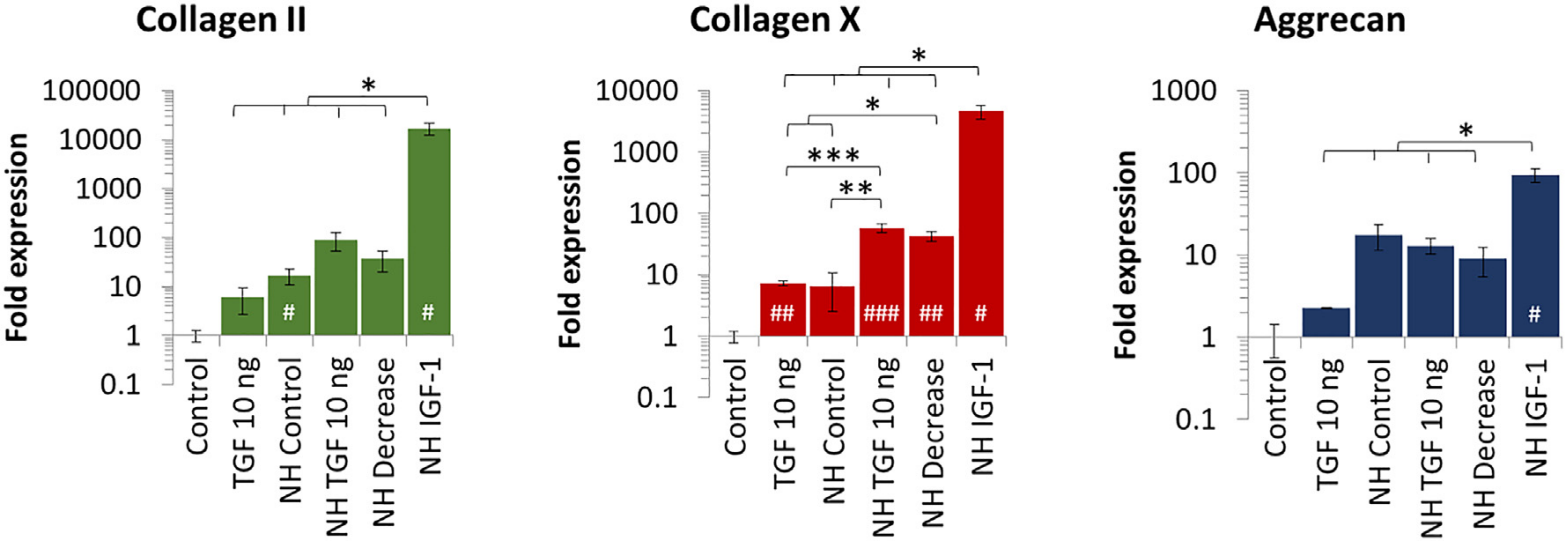
Compensation of negative effects coming from a high dose of nanoparticles using culture parameters. MSCs labeled with a high dose of nanoparticles (> 30 pg/cell) are differentiated for 27 days using varying growth factors supplementation patterns (described in Fig. 2) and oxygen tension (NH represents the normoxic-hypoxic culture, with 7 days under normoxia followed by 20 days under hypoxia). Gene expression is assessed at day 27. With these highly labeled cells, the NH TGF 10 ng condition induces only limited collagen II and aggrecan expression, while it drove high collagen II and aggrecan expression with unlabeled cells. Culture under NH with TGF-β3 at 10 ng/mL and IGF-1 at 100 ng/mL (NH IGF-1) highly increases collagen II and aggrecan expression compared to the other conditions. However, collagen X is also upregulated. Expression was normalized to RPLP0 mRNA and expressed relative to average value of unlabeled cells cultured without growth factor supplement under normoxia (control). Significance between groups was determined using independent Student's *t*-test. # represents significant differences when compared to the control. * represents significant differences between conditions.

Magnetic nanoparticles are used in numerous biomedical applications. Their composition is based on an iron core that is progressively assimilated by the natural iron metabolism after internalization in cells. However, at a high dose of nanoparticles (> 30 pg iron/cell) chondrogenesis is clearly inhibited, as observed in Fig. 7 and previously reported [38]. Herein, we aimed to assess whether such negative effects of the nanoparticles under chondrogenesis can be countered. Using selected culture conditions that gave improved results in the previous experiments, we first observe that chondrogenesis is strongly reduced for most conditions at day 27 (Fig. 8). A NH conditioning with 10 ng/mL of TGF-β3 led to a significant decrease in collagen II and aggrecan expression when compared to unlabeled cells. Similarly, collagen X expression was also decreased. Nevertheless, when cells were cultured under NH, with 10 ng/mL of TGF-β3, and 100 ng/mL of IGF-1, it significantly reduced the detrimental effects of high dose labeling at day 27, with values of collagen II and aggrecan expression close to those obtained without nanoparticles.

**Fig. 9:**
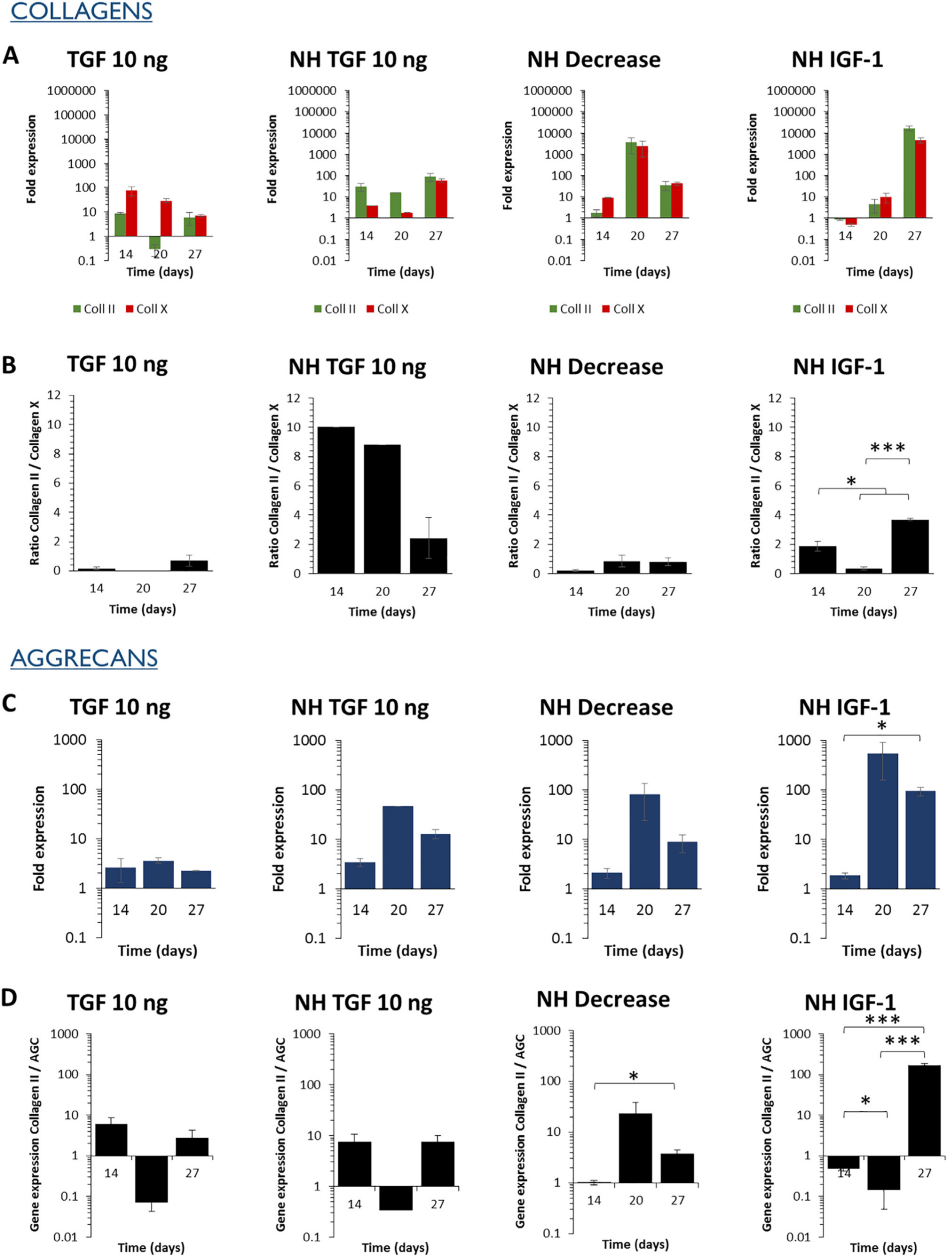
Impact of the culture microenvironment on the chondrogenic potential of stem cells labeled with a high dose of magnetic nanoparticles. MSCs are labeled with a high dose of nanoparticles (> 30 pg/cell), then 27 days of chondrogenic differentiation is induced using varying growth factors supplementation patterns (described in Fig. 2) and oxygen tension (NH represents the normoxic-hypoxic culture, with 7 days under normoxia followed by 20 days under hypoxia). (A) The gene expression of collagen II as well as collagen X is assessed and their statistical significance is displayed in Table S5. (B) The collagen II-to-collagen X ratio is calculated and used as an indicator of hypertrophy level. (C) Aggrecan gene expression, typical ECM component of hyaline cartilage is assessed. (D) The collagen II-to-aggrecan ratio is calculated to ensure that an important secretion of aggrecan does not have a negative impact on the expression of collagen II. Expression was normalized to RPLP0 mRNA and expressed relative to average value of unlabeled cells cultured without growth factor supplement under normoxia (control). Significance between groups was determined using independent Student's *t*-test. # represents significant differences when compared to the control. * represents significant differences between conditions.

Moreover, the kinetics over days 14, 20, and 27 have shown that the highest expression levels of collagen II are delayed (Fig. 9). Indeed, the increased expression of collagen II, aggrecan, and improved collagen II-to-collagen X and collagen II-to-aggrecan ratios were obtained at day 27 only (NH IGF-1 condition).

## 4 Discussion

In current research, factors playing a role in cartilage development have been identified; however, the impact of fine stimulation of these factors on chondrogenic differentiation still remains unclear. As chondrogenesis in vitro is most often performed under normoxic conditions, we first studied the influence of TGF-β3 and IGF-1 supplementation patterns on the differentiation of MSCs in normoxia. It has been suggested that punctual supplementation of TGF-β3 might be preferential [41]. However, we here demonstrate that a continuous addition of TGF-β3 at an above threshold concentration (10 ng/mL) is actually more efficient in terms of collagen II and aggrecan expression as well as to limit collagen X production. An abundant expression of aggrecan and collagen II is indeed observed and indicates that the network of collagen II is already started before proteoglycans' synthesis, as excessively rapid initial proteoglycans' synthesis impedes collagen II synthesis [41]. In an effort to further improve these results, the TGF-β3 10 ng/mL condition was supplemented with IGF-1 at 100 ng/mL. It has previously been shown that IGF-1 improves differentiation, maintains the chondrocyte phenotype, and prevents cells from progressing to the hypertrophic stage [22,43]. Here, no additive effect was observed in the presence of IGF-1 when compared with TGF-β3 alone.

Interestingly, under the best condition (TGF-β3 at 10 ng/mL), the highest collagen II and aggrecan expression was at day 20 as well as the highest collagen II-to-collagen X and collagen II-to-aggrecan ratios. It suggests that the chondrogenesis might be too advanced at day 27, usual time for final chondrogenic differentiation in vitro.

As these normoxic cultures do not fully represent the native environment, effects of hypoxia were then assessed. Indeed, the cartilage tissue is avascular, thus oxygen diffusion is limited and the tissue presents various levels of hypoxia depending on the distance from the synovial fluid (7 to 1% O_2_) [23,24]. Previous studies have shown hypoxic stimulation is a strong promoter of matrix deposition [[44], [45], [46], [47]]. We here observed that culture under transient hypoxia is actually more efficient than continuous hypoxia. Cells cultured under 21% O_2_ for 7 days followed by 3% O_2_ for 20 days presented increased collagen II and aggrecan expression compared to cells under continuous hypoxia (3% O_2_). Consistent with our findings, Gómez-Leduc et al. made similar observations for the differentiation of umbilical cord mesenchymal stem cells as chondrocytes [48]. Under this normoxic-hypoxic (NH) conditioning, chondrogenesis was initiated even without the use of growth factors. It has been described on several cell models that hypoxia promotes the expression of various hypoxia inducible factors (HIF) that are known to influence chondrogenesis [49,50]. It is thus not surprising that hypoxia alone can be an initiator of chondrogenesis. The effect of hypoxia was amplified in the presence of growth factors, such as a continuous add of TGF-β3 (10 ng/mL) that presented highest collagen II and aggrecan expression as well as improved collagen II-to-collagen X ratios (compared to normoxia).

Interestingly, the synthesis of ECM components was faster under NH than normoxia with an advanced expression at day 14 under a continuous add of TGF-β3. It suggests that chondrogenesis should be stopped at day 14 to limit further hypertrophy.

Overall, under both normoxic and normoxic-hypoxic culture, chondrogenesis seems too advanced at day 27 driving differentiated MSCs toward the hypertrophic phenotype as indicated by increased collagen X expression and reduced collagen II-to-collagen X ratio. It then seems more judicious to stop the differentiation earlier, around of day 21 under normoxia and day 14 under normoxia-hypoxia. Indeed, hypoxia increased the kinetics of collagen II and aggrecan expression indicating a faster and stronger differentiation.

The use of magnetic nanoparticles in medicine opens up numerous possibilities for regenerative medicine as they can allow for cell tracking upon implantation by means of MRI, tissue development, or cell stimulation. Low doses of nanoparticles have been shown not to impact stem cell differentiation; however, at high dose a clear impact is denoted with a reduced expression of collagen II and aggrecans [38]. We demonstrate here that it is possible to counter the negative effects coming from a high nanoparticles' dose with a combination of hypoxia, TGF-β3 and IGF-1. Indeed, by subjecting the cells to hypoxia when cultured with TGF-β3 and IGF-1, collagen and aggrecan synthesis is strongly increased at day 27. However, nanoparticles still delayed chondrogenesis with a very low expression of collagen II and aggrecan at day 14 (compared to the same conditions without nanoparticles) and starting to be expressed at day 20 only. The positive effect of hypoxia and IGF-1 might be linked to their antioxidant activity. Indeed, nanoparticles internalized in stem cells and cultured under a chondrogenic model are progressively degraded overtime and release free iron ions that are progressively stored in the iron storage protein ferritin [35]. However, the free iron released can engender the production of ROS via the Fenton reaction; when ROS production exceeds the antioxidant capacities of the cell, an “oxidative stress” occurs leading to structural and functional cartilage damages [51]. With their antioxidant activity, hypoxia and IGF-1 might be able to counter this Fenton reaction thus the negative effects of the nanoparticles. The protector effect of IGF-1 against ROS might acts via the enhancement of the activity of antioxidant enzymes such as the glutathione peroxidase (GPX) [52]. In any case, the internalization of nanoparticles in stem cells, very promising in regenerative medicine, should be closely monitored and controlled in order to have a fast and efficient chondrogenesis.

To conclude, we observed that the best conditions to drive MSC differentiation such as expressing hyaline cartilage markers was to initiate chondrogenesis under normoxia and pursue the differentiation under hypoxia, with a continuous supplementation of TGF-β3 at 10 ng/mL. This normoxic-hypoxic culture setup, despite being more demanding in terms of materials needed and experimental design, increases gene expression of collagen II and aggrecan and limits expression of the hypertrophic marker collagen X relatively to collagen II. Besides, we showed that the negative effects of a high-dose of nanoparticles could be overcome by specific culture conditions consisting of the normoxic-hypoxic culture combined with TGF-β3 and IGF-1 supplementation. Overall, we have shown that the usual duration of chondrogenesis in vitro, 27 days, is too long. These results should be taken into consideration to adjust culture strategies for regenerative medicine applications and further tissue implantation.

## Competing interests

The authors declare no competing financial interests.

## Declaration of interest form

The authors declare no conflict of interest related to this work.

## Appendix A Supplementary data

Supplementary tables Image 1

Supplementary figures Image 2
