# Supplementary material for: Role of growth factors and oxygen to limit hypertrophy and impact of high magnetic nanoparticles dose during stem cell chondrogenesis
Source: Comput Struct Biotechnol J. 2018 Oct 30;16:532–42. doi: 10.1016/j.csbj.2018.10.014 (PMC6260287; doi:10.1016/j.csbj.2018.10.014)
Supplement: Supplementary file 1 — Supplementary tables [file mmc1.docx]

**Supplementary tables**

**Table S1: Materials list**

| Name of Material/ Equipment | Provider | Catalog Number |
| --- | --- | --- |
| 0.05% Trypsin-EDTA (1x) | Life Technologies | 25300-054 |
| Dexamethasone | Sigma | D4902 |
| DMEM with Glutamax I | Life Technologies | 31966-021 |
| Ethanol absolute | VWR | 20821.310 |
| Formalin solution 10% neutral buffered | Sigma | HT5012 |
| IGF-1 protein | R&D Systems | 291-G1 |
| Iron oxide (maghemite) nanoparticules (γ-Fe_2_O_3_) | PHENIX - University Paris 6 | Made and given by C. Ménager |
| Isopentane | Sigma | M32631 |
| ITS Premix Universal Culture Supplement (20x) | Corning | 354352 |
| L-Ascorbic Acid 2-phosphate | Sigma | A8960 |
| L-Proline | Sigma | P5607 |
| Mesenchymal Stem Cell (MSC) | Lonza | PT-2501 |
| Mounting medium Pertex | Histolab | 840 |
| MSCGM BulletKit medium | Lonza | PT-3001 |
| NucleoSpin RNA Extraction Kit | Macherey-Nagel | 740955.5 |
| OCT solution | VWR | 361603E |
| PBS w/o CaCl_2_ w/o MgCl_2_ | Life Technologies | 14190-094 |
| PCR nucleotide dNTP mix (10mM each) | Roche | 10842321 |
| Penicillin (10.000U/mL)/Streptomicin (10.000µg/mL) | Life Technologies | 15140-122 |
| Random Primer - Hexamer | Promega | C1181 |
| Recombinant RNAsin ribonuclease inhibitor | Promega | N2511 |
| RPMI medium 1640, no Glutamine | Life Technologies | 31870-025 |
| Sodium pyruvate solution 100mM | Sigma | S8636 |
| Step One Plus Real-Time PCR System | Life Technologies | 4381792 |
| SuperScript II Reverse Transcriptase | Life Technologies | 18064-014 |
| SyBr Green PCR Master Mix | Life Technologies | 4368708 |
| TGF-beta 3 protein 10µg | Interchim | 30R-AT028 |
| Toluene | VWR | 1.08323.1000 |
| Toluidine blue O | VWR | 1.15930.0025 |
| Tri-sodium citrate | VWR | 33615.268 |

**Table S2: List of primer sequences for gene expression analyses by quantitative real-time PCR.**

| Gene | Accession number | Sense Primer | Antisense Primer |
| --- | --- | --- | --- |
| RPLP0 | NM_053275.3 | 5’-TGCATCAGTACCCCATTCTATCA-3’ | 5’-AAGGTGTAATCCGTCTCCACAGA-3’ |
| ACAN | NM_001135.3 | 5’-TCTACCGCTGCGAGGTGAT-3’ | 5’-TGTAATGGAACACGATGCCTTT-3’ |
| Col2A1 | NM_001844.4 | 5’-ACTGGATTGACCCCAACCAA-3’ | 5’-TCCATGTTGCAGAAAACCTTCA-3’ |
| Col10A1 | NM_000493.3 | 5’-TGCCCACAGGCATAAAAGG-3’ | 5’-TTTATGGTGTAGGGAATGAAGAACTG-3’ |

RPLP0: Ribosomal protein large subunit P0 ; ACAN: Aggrecan; Col2A1: Collagen type II; Col10A1: Collagen type X.

**Table S3: Statistical analysis of collagen II and collagen X expression under normoxia.** A T-test is performed between experimental days to determine significance of the results presented in Figure 4.

|  |  | **P-value** | |
| --- | --- | --- | --- |
|  | **Days** | **collagen II** | **collagen X** |
| **TGF 1 ng** | **7-14** | 0.1079 | 0.0179 |
|  | **7-20** | 0.1091 | 0.0245 |
|  | **7-27** | 0.2229 | 0.0064 |
|  | **14-20** | 0.1265 | 0.1984 |
|  | **14-27** | 0.3987 | 0.5143 |
|  | **20-27** | 0.4457 | 0.3214 |
| **TGF 10 ng** | **7-14** | 0.0138 | 0.0238 |
|  | **7-20** | 0.0054 | 0.0190 |
|  | **7-27** | 0.0450 | 0.0178 |
|  | **14-20** | 0.0085 | 0.0426 |
|  | **14-27** | 0.8265 | 0.1735 |
|  | **20-27** | 0.0094 | 0.1051 |
| **Pulse** | **7-14** | 0.0066 | 0.0001 |
|  | **7-20** | 0.0001 | 0.0037 |
|  | **7-27** | 0.0461 | 0.0035 |
|  | **14-20** | 0.0267 | 0.0009 |
|  | **14-27** | 0.0082 | 0.0029 |
|  | **20-27** | 0.0007 | 0.2547 |
| **Decrease** | **7-14** | 0.0008 | 0.0006 |
|  | **7-20** | 0.0029 | 0.0015 |
|  | **7-27** | 0.1237 | 0.0039 |
|  | **14-20** | 0.0090 | 0.0115 |
|  | **14-27** | 0.0025 | 0.0448 |
|  | **20-27** | 0.0382 | 0.3838 |
| **IGF-1** | **7-14** | 0.0083 | 0.0257 |
|  | **7-20** | 0.1157 | 0.1915 |
|  | **7-27** | 0.0506 | 0.0271 |
|  | **14-20** | 0.2248 | 0.2814 |
|  | **14-27** | 0.3046 | 0.5815 |
|  | **20-27** | 0.1777 | 0.3143 |

**Table S4: Statistical analysis of collagen II and collagen X expression under NH.** A T-test is performed between experimental days to determine significance of the results presented in Figure 6.

|  |  | **P-value** | |
| --- | --- | --- | --- |
|  | **Days** | **collagen II** | **collagen X** |
| **NH TGF 1 ng** | **7-14** | 0.0000 | 0.0008 |
|  | **7-27** | 0.2082 | 0.0601 |
|  | **14-27** | 0.3322 | 0.1274 |
| **NH TGF 1 ng** | **7-14** | 0.0345 | 0.0623 |
|  | **7-20** | 0.1111 | 0.1086 |
|  | **7-27** | 0.0400 | 0.0111 |
|  | **14-20** | 0.4277 | 0.1599 |
|  | **14-27** | 0.0681 | 0.0140 |
|  | **20-27** | 0.1271 | 0.1078 |
| **NH Pulse** | **7-14** | 0.2961 | 0.3592 |
|  | **7-20** | 0.0977 | 0.0424 |
|  | **7-27** | 0.0727 | 0.0157 |
|  | **14-20** | 0.5756 | 0.3459 |
|  | **14-27** | 0.6206 | 0.1686 |
|  | **20-27** | 0.8512 | 0.5444 |
| **NH Decrease** | **7-14** | 0.0233 | 0.0233 |
|  | **7-20** | 0.1138 | 0.1183 |
|  | **7-27** | 0.0001 | 0.0017 |
|  | **14-20** | 0.5740 | 0.3198 |
|  | **14-27** | 0.0001 | 0.0024 |
|  | **20-27** | 0.0005 | 0.0064 |
| **NH IGF-1** | **7-14** | 0.1050 | 0.0929 |
|  | **7-20** | 0.0288 | 0.0621 |
|  | **7-27** | 0.1414 | 0.0946 |
|  | **14-20** | 0.0695 | 0.0895 |
|  | **14-27** | 0.1474 | 0.1059 |
|  | **20-27** | 0.1715 | 0.2587 |

**Table S5: Statistical analysis of collagen II and collagen X expression for nanoparticles’ labeled MSCs.** A T-test is performed between experimental days to determine significance of the results presented in Figure 9.

|  |  | **P-value** | |
| --- | --- | --- | --- |
|  | **Days** | **collagen II** | **collagen X** |
| **TGF 10 ng** | **14-20** | 0.000141652 | 0.1408058 |
|  | **14-27** | 0.369939822 | 0.0497088 |
|  | **20-27** | 0.103689282 | 0.0271522 |
| **NH TGF 10 ng** | **14-20** | - | - |
|  | **14-27** | 0.546411505 | - |
|  | **20-27** | - | - |
| **NH Decrease** | **14-20** | 0.158169474 | 0.1579193 |
|  | **14-27** | 0.171285515 | 0.0208649 |
|  | **20-27** | 0.161290733 | 0.1623782 |
| **NH IGF-1** | **14-20** | 0.185266875 | 0.0780678 |
|  | **14-27** | 0.033513347 | 0.0319648 |
|  | **20-27** | 0.033535678 | 0.0321665 |
