# Supplementary material for: Role of growth factors and oxygen to limit hypertrophy and impact of high magnetic nanoparticles dose during stem cell chondrogenesis
Source: Comput Struct Biotechnol J. 2018 Oct 30;16:532–42. doi: 10.1016/j.csbj.2018.10.014 (PMC6260287; doi:10.1016/j.csbj.2018.10.014)
Supplement: Supplementary file 2 — Supplementary figures [file mmc2.docx]

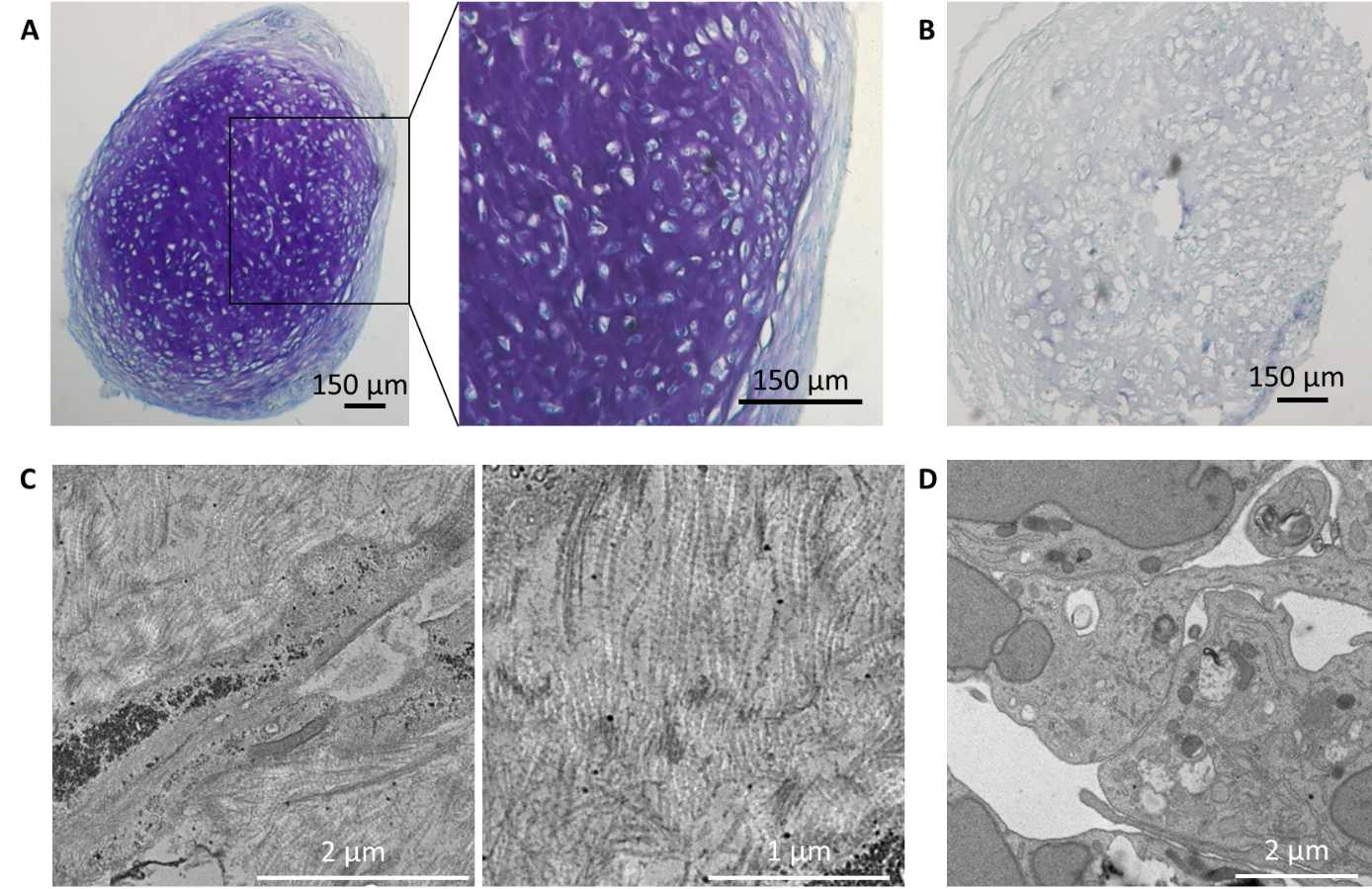


**Figure S1: Proteoglycans and collagen II deposition upon chondrogenic differentiation under normoxia and a continuous supplementation with TGF-β3 (10 ng/mL).** MSCs non labeled with magnetic nanoparticles were differentiated for 27 days (under TGF 10 ng conditioning) and compared with the negative control (no growth factors added). At day 27, proteoglycans and collagen II deposition were assessed via staining and transmission electron microscopy (TEM), respectively. (A-B) Sections of differentiated (A) or undifferentiated (B) spheroids were stained with toluidine blue. The deep blue color present in the differentiated spheroids indicated the presence of proteoglycans, while none were observed in the control spheroids. (C-D) TEM images of differentiated (C) or undifferentiated (D) spheroids were taken at day 27. The differentiated spheroids displayed an important secretion of extracellular matrix and the presence of collagen II depicted by a periodicity in the collagen fibers clearly seen on the middle image, while no extracellular matrix was observed for the negative control (right image).


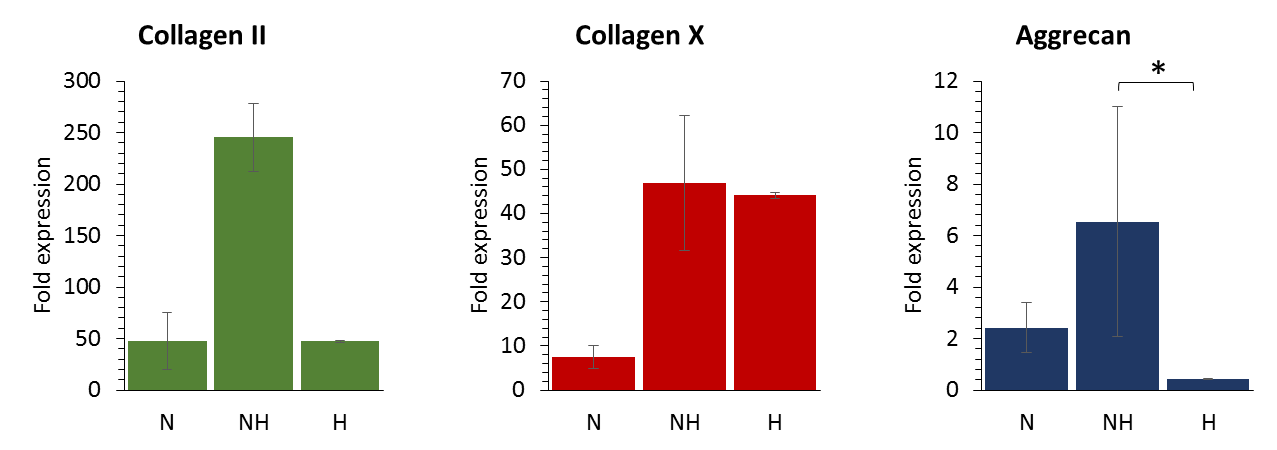


**Figure S2: Nuances in oxygen levels influence chondrogenesis.** A 27 days chondrogenic differentiation is performed under continuous normoxia (N), under 7 days of normoxia followed by 20 days of hypoxia (NH), or continuous hypoxia (H) (normoxia: 21% O_2_, hypoxia: 3% O_2_). Results at day 27 display significantly higher collagen II, collagen X, and aggrecan under NH, while only collagen X expression is increased under H. For this experiment, chondrogenesis is performed on MSCs labeled with a high dose of nanoparticles (> 30 pg/cell). Expression was normalized to RPLP0 mRNA and expressed relative to average value of cells cultured without growth factor supplement and cultured under normoxia (control). Significance between groups was determined using independent Student’s *t*-test. # represents significant differences when compared to the control. * represents significant differences between conditions.
